# Neural Signatures of Performance Feedback in the Paced Auditory Serial Addition Task (PASAT): An ERP Study

**DOI:** 10.3389/fnhum.2021.630468

**Published:** 2021-02-18

**Authors:** Anja Sommer, Lukas Ecker, Christian Plewnia

**Affiliations:** Department of Psychiatry and Psychotherapy, Neurophysiology & Interventional Neuropsychiatry, University Hospital Tübingen, Tübingen, Germany

**Keywords:** cognitive control, PASAT, event related potential, cognitive control training, negative information processing

## Abstract

Research on cognitive control has sparked increasing interest in recent years, as it is an important prerequisite for goal oriented human behavior. The paced auditory serial addition task (PASAT) has been used to test and train cognitive control functions. This adaptive, challenging task includes continuous performance feedback. Therefore, additional cognitive control capacities are required to process this information along with the already high task-load. The underlying neural mechanisms, however, are still unclear. To explore the neural signatures of the PASAT and particularly the processing of distractive feedback information, feedback locked event-related potentials were derived from 24 healthy participants during an adaptive 2-back version of the PASAT. Larger neural activation after negative feedback was found for feedback related negativity (FRN), P300, and late positive potential (LPP). In early stages of feedback processing (i.e., FRN), a larger difference between positive and negative feedback responses was associated with poorer overall performance. This association was inverted in later stages (i.e., P300 and LPP). Together, our findings indicate stage-dependent associations between neural activation after negative information and cognitive functioning. Conceivably, increased early responses to negative feedback signify distraction, whereas higher activity at later stages reflects cognitive control processes to preserve ongoing performance.

## Introduction

In a world full of competing information and sources of distraction, the ability to maintain coordinated and purposeful behavior is essential to sustain goal directed processes. This requires cognitive control, which comprises different cognitive functions including the ability to pay selective attention, ignore distracting information, turn attention away from stimuli when they prove irrelevant, and the ability to store and manipulate internal representations of information (Roiser et al., 2012). Especially the inhibition of irrelevant but salient information, like emotional stimuli challenges cognitive control (Iordan et al., 2013). Cognitive control is a key factor for successful human behavior. Therefore, it is not surprising that dysfunctional cognitive control is increasingly recognized as a key feature of various psychiatric disorders. In fact, research shows that in particular patients suffering from depression are prone to a heightened sensitivity toward negative stimuli, which receive more attention and working memory capacity and therefore impede the maintenance of coordinated and purposeful behavior (De Raedt and Koster, 2010). This “negativity bias” constitutes an important factor for the development and maintenance of depression as well as a central mechanism of recovery via restoration of cognitive control functioning (Roiser et al., 2012). Consistently, impairment of goal-directed behavior can be observed in healthy participants when cognitive resources are occupied by emotionally salient distractors (Dolcos and McCarthy, 2006).

A task used to investigate cognitive control is the “paced auditory serial addition task” (PASAT) (Gronwall, 1977) in which digits are presented auditorily and participants add the current digit to the digit they heard before. In its adaptive version, inter-stimulus intervals (ISI) decrease (increase) when several consecutive trials are correct (incorrect). The PASAT has been used as a cognitive control task in healthy (Siegle et al., 2014; Plewnia et al., 2015; Pope et al., 2015; Wiegand et al., 2019) as well as clinically depressed (Hoorelbeke et al., 2015; Koster et al., 2017) and at-risk participants (Van den Bergh et al., 2018). These comprehensive data indicate that this task is particularly suitable to investigate and train cognitive control in both, healthy subjects and psychiatric patients. Regarding its specific mechanism, it has been shown that the PASAT induces frustration and negative affect presumably due to receiving continuously feedback on current performance and working at the individual processing speed limit. Furthermore, the negative affective change induced by the PASAT can be correlated with a lower performance (Plewnia et al., 2015), implying that task- or feedback-related irritation must be sufficiently compensated to uphold goal directed behavior. Therefore, the PASAT challenges cognitive control by means of emotional and cognitive responses to feedback information at a high cognitive load. On a neurophysiological level, prefrontal control on limbic areas plays a key role in overcoming this distraction caused by negative information (Plewnia et al., 2015). The dorsolateral prefrontal cortex (dlPFC) influences PASAT performance (Lazeron et al., 2003). However, more studies are needed for a better understanding of the mechanisms of action underlying the PASAT.

Therefore, the goal of the current study is to investigate the highly time dynamic neural signatures of the PASAT. Due to their high temporal resolution event related potentials (ERP) are best suited to find such neural signatures of PASAT performance. To capture the conflict of competing negative information and ongoing cognitive functioning in the PASAT, ERPs locked to performance feedback presented simultaneously with the next target digit, are derived from three different processing stages.

The feedback related negativity (FRN) is a negative deflection having its peak between 200 and 300 ms after a performance feedback stimulus was presented. It is an ERP that has been shown to be sensitive to feedback valance (Ridderinkhof et al., 2004; Hajcak et al., 2006). It is larger for negative than positive feedback and maximal at medial frontal electrode sites (Gehring and Willoughby, 2002). Besides of its informative value concerning current task performance (Holroyd and Coles, 2002; Holroyd and Yeung, 2012) it has been suggested that the FRN indicates the emotional impact of a negative expectation violation (Luu et al., 2003), implying that feedback does involve emotional processing that captures cognitive resources. Since in our study negative information is operationalized by the given feedback, we utilized the FRN to investigate early parts of negative information processing. Of note, in our study its registration conditions, however, differ from the common investigations of the FRN or feedback processing because it summarizes the processing of new information (next digit) and performance feedback (last digit).

Attention allocation to task relevant as well as subsequent memory processes is reflected by the P300. It is a positive deflection peaking between 300 and 400 ms following stimulus presentation which is maximal at midline-parietal sites (Sutton et al., 1965; Polich, 2012). Furthermore, an enhanced amplitude for emotional compared to neutral stimuli can be observed for both, positive and negative content (Johnston et al., 1986; Keil et al., 2002; Delplanque et al., 2004) probably reflecting high inherent motivational salience of emotional stimuli *per se*. Therefore, it seems to be best suited to study negative information processing during a demanding cognitive task. Moreover, several studies have linked larger P300 amplitudes with performance gains in non-emotional (Daffner et al., 2011; Saliasi et al., 2013) as well as in emotional tasks (Palomba et al., 1997) making it suitable to investigate associations of neural feedback processing and PASAT performance in our study.

The late positive potential (LPP) is known to capture attention allocation toward emotional salient stimuli (Cacioppo et al., 1996; Ito et al., 1998). It is recorded at centro-parietal sites and begins as early as 200–300 ms post-stimulus. In contrast to the P300 it can outlast the stimulus presentation well beyond several seconds (Hajcak et al., 2009). Therefore, besides its sensitivity to automatic attention allocation to emotional stimuli, it reflects continued processing of emotional content and is regulated by top-down mechanisms. Moreover, the magnitude of the LPP amplitude has also been linked to task performance (Weinberg and Hajcak, 2011; Bamford et al., 2015; Faehling and Plewnia, 2016). We want to utilize the LPP in our study to capture late neural reactions to negative information in the form of feedback and moreover to investigate its associations with the PASAT performance.

Taken together, with this study, we investigate the time dynamic neural signatures (FRN, P300, and LPP) of the PASAT and aim for a better understanding of the underlying mechanisms. As negative feedback is associated with negative affect and competes with ongoing performance, we assume to find larger amplitudes for negative than positive feedback in all stages of feedback processing. Furthermore, we hypothesize to find significant correlations of PASAT performance and ERP magnitudes indicating associations of cognitive control functioning and neural activation.

## Materials and Methods

### Subjects

Twenty-five healthy participants were recruited via internet advertisement. All participants had normal or corrected to normal vision and normal hearing. Exclusion criteria were current psychiatric disorders, neurological disorders, major head injuries or color blindness. They received a financial compensation or course credit for their participation. All participants gave their written informed consent. One participant had to be excluded due to excessive noise in the electroencephalographic (EEG) data (see section “Electrophysiological Data Processing”). The remaining 24 participants (16 female, age: *M* = 23.71, *SD* = 4.06) were included in the analysis. See Supplementary File 1 for the data underlying the sample characteristics and Table 1 of the Supplementary Materials for demographic and neuropsychological characteristics of the sample. The study was approved by the Ethics Committee of the Medical Faculty of the Eberhard-Karls-University and at the University Hospital Tübingen and was conducted in compliance with the Declaration of Helsinki.

### Tasks

The tasks *PASAT, color presentation* and *feedback-neutral PASAT* outlined below were computer-based and implemented using PsychoPy2 v1.80.02 (Peirce, 2007, 2008). They were presented on a 17-inch monitor.

#### Paced Auditory Serial Addition Task (PASAT)

We used a 2-back version of an adaptive Paced Auditory Serial Addition Task (PASAT). Participants sat in front of a monitor (distance: approximately 65 cm) and heard digits (1–9, duration of presentation: 433–567 ms) via in ear headphones. The task was to add the current digit to the digit they heard before the last one (2-back). Results were indicated by pressing the corresponding key on a keyboard that was equipped with the response letters 2–18. Feedback was given after each trial simultaneously with the presentation of the new digit by presenting green (red) light after correct (incorrect) responses. In order to make the feedback highly salient the whole monitor was filled with the corresponding feedback color (e.g., 17 inch). The duration of the feedback presentation was 433 ms (matched to the presentation duration of the shortest number). Initially the ISI was set to 3 s. The ISI thereby refers to the time in between presented digits as well as feedback, since it was presented simultaneously. The ISI was decreased (increased) after four consecutively correct (incorrect) trials by 100 ms. This causes the PASAT to adapt to the capability of each participant while remaining challenging. The task comprised three blocks each with a duration of 5 min with 30 s of break in between. The total number of correct trials was used as the main outcome variable. Because the PASAT is highly demanding both to WM and processing speed, it is challenging to stay focused throughout the duration of the task and to not get distracted by the feedback provided. According to our hypothesis, lack in cognitive control would result in fewer consecutive correct responses. Therefore, we calculated the proportion of consecutive correct relative to the overall correct responses as a second outcome variable (subsequently referred to as “performance stability”). Moreover, for the ERPs of the PASAT, only feedback following a response was used (e.g., trials with red feedback for a missing response were excluded from analysis).

#### Control Task “Color Presentation”

Since we aimed to test the differential neural responses to feedback valence as indicated by red and green screen color in contrast to the neural activation to red and green color as such, we conducted a control task “color presentation” (CP). Participants were asked to sit in front of a monitor (distance: approximately 65 cm) and perceive red and green light peripheral by keeping their gaze on the keyboard just like they would do while performing the PASAT. The task consisted of two blocks each with a duration of 2.5 min. Red and green light was presented for 433 ms (as in the PASAT) in random order with a jittered inter stimulus interval (1,500–2,500 ms).

#### Control Task “Feedback-Neutral PASAT”

Since differences in neural activity for negative feedback from positive feedback could be due to error monitoring based on the mistake and not the processing of the negative performance feedback as such, we additionally conducted a feedback neutral version of the PASAT (“feedback-neutral PASAT”). The procedure of this feedback-neutral PASAT was exactly like for the PASAT (see section “Paced Auditory Serial Addition Task (PASAT)”), except that no feedback was presented. Furthermore, the feedback-neutral PASAT only comprised two blocks of 5 min each.

### Electroencephalography Recording

The electroencephalogram (EEG) was recorded using an elastic cap (EASYCAP GmbH, Herrsching, Germany), the actiCHamp amplifier system with 32 active Ag/AgCl electrodes and the corresponding Brain Vision Recorder system (Brain Products GmbH, Gilching, Germany). EEG was registered from 27 scalp sites (FP1, F7, F3, Fz, F4, F8, FC5, FC1, FCz, FC2, FC6, C3, Cz, C4, CP5, CP1, CPz, CP2, CP6, P7, P3, Pz, P4, P8, O1, Oz, O2). Additionally, an electrooculogram (EOG) was recorded. For horizontal eye movements two electrodes were placed approximately one cm left and right of the eyes. One electrode positioned approximately one cm below the left eye and the Fp1 electrode were used to register vertical eye movements. Furthermore, electrodes were placed on the left and right mastoid. The left mastoid served as the online reference and a forehead electrode as the ground. The online sampling rate was 1,000 Hz. Impedances were kept below 10 kΩ before initiation of the recording. To check if time locking of the EEG trace and events was correct, we additionally equipped the presentation monitor with a photo sensor (Brain Products GmbH, Gilching, Germany). There were no time differences between the signals.

### Procedure

The experiment took place in a dimly lit, quiet room. After the participants gave their written informed consent, the EEG electrodes were attached to the scalp. Participants were asked to sit quietly during the EEG recording. For all participants the experiment started with the CP before the PASAT was conducted. Although this could lead to a confounding of the data by order effects, we refrained from balancing the order of the two tasks since we feared that if participants performed the CP task after the PASAT they would have learned the specific association of color and feedback. Deriving neural signatures of the mere color presentation, which was the goal of the CP, would thus not be possible anymore as the signal would be confounded by learned associations and it can be assumed that this would lead to a stronger confound of the data than a non-mixed task order. After the CP, participants carried out the PASAT. To make sure participants understood the instruction of the task, they completed 30 practice trials, which were excluded from analysis. To control for affective effects of the PASAT, participants completed the 20 item positive and negative affect schedule (Krohne et al., 1996) immediately before and after the PASAT. That followed a resting phase of 7 min during that heart rate measures were obtained, which are not subject of the current paper. Afterward participants completed the control task “feedback-neutral PASAT.”

### Electrophysiological Data Processing

We analyzed the EEG data using the EEGLAB toolbox (Delorme and Makeig, 2004) running on MATLAB 9.2 R2017a (The MathWorks, Natick, MA, United States) and the EEGLAB toolbox ERPLAB (Lopez-Calderon and Luck, 2014). The raw EEG was resampled offline to 250 Hz and re-referenced to an average of the left and right mastoids. Band-pass filters with a low and high cutoff of 0.1 and 35 Hz, and a notch-filter at 50 Hz were applied. Ocular artifacts were removed manually using independent component analysis (JADE algorithm; Cardoso, 1999). Subsequently feedback locked epochs were extracted ranging from −100 to 1,000 ms relative to feedback (PASAT), color (CP), respectively. For the feedback-neutral PASAT the epochs were locked to the digit presentation, which is exactly the same time the feedback would have been presented in the PASAT. Just like for the PASAT and CP, epochs of the feedback-neutral PASAT ranged from −100 to 1,000. Artifact correction was conducted in the epoched EEG. Epochs containing EEG signals exceeding an amplitude of 65 μV within a 100 ms moving window or exceeding −65 to +65 μV within the epoch were considered artifacts and were rejected (using the ERPLAB implemented automated artifact detection). Participants with more than 25% of rejected epochs were excluded from further analysis (*n* = 1). In the PASAT on average *M* = 4.12% of the green feedback trials (*SD* = 5.50%) and *M* = 4.60% of the red feedback trials (*SD* = 5.27%) were rejected. In the CP *M* = 2.57% of the green color trials (*SD* = 4.67%) and *M* = 3.25% of the red color trials (*SD* = 6.76%) were rejected. In the feedback-neutral PASAT on average *M* = 3.98% of the correct trials (*SD* = 6.53%) and *M* = 5.62% of the incorrect trials (*SD* = 7.83%) were rejected. Overall, 2,610 green and 1,245 red feedbacks of the PASAT, 1,796 green and 1,771 red color trials and 1,874 correct and 1,467 incorrect trials of the feedback-neutral PASAT were included in the ERP analysis. In sum, there were six conditions for the calculation of the ERPs: green color after a correct trial in the PASAT (green feedback), red color after an incorrect trial in the PASAT (red feedback), green color in the CP, red color in the CP, incorrect trials in the feedback-neutral PASAT and correct trials in the feedback-neutral PASAT. ERPs for the analysis of the PASAT results were constructed by separately averaging trials in the four conditions (green feedback, red feedback, green color, red color). Subsequently we calculated difference waves: positive feedback = green feedback (PASAT) - green color (CP), negative feedback = red feedback (PASAT) – red color (CP). All further ERP analyses refer to these difference waves (see Supplementary Figures 1–6 in the Supplementary Materials depicting the raw waveforms and scalp maps separately for the PASAT and CP for the FRN, P300 and LPP). ERPs for the analysis of the feedback-neutral PASAT were constructed by separately averaging trials in the two conditions correct and incorrect trials.

We chose the electrode sites and time windows to measure the FRN, P300, and LPP according to previous literature. The FRN was defined as the mean amplitude within a time window between 200 and 300 ms following feedback at Fz (Gehring and Willoughby, 2002). The P300 was scored as the average of three centro-parietal sites (Cz, CPz, Pz) (Sutton et al., 1965; Johnson, 1993). According to visual inspection there is a large shift in the P300 waveforms due to the FRN (see Figure 2). Therefore, we defined the P300 as the averaged ERP waveform for each participant as the base-to-peak difference in voltage between the most negative peak between 200 and 300 ms post feedback and the most positive peak 300–400 ms post feedback (Fabiani et al., 1987; Polich, 1991; Polich and Kok, 1995). The LPP was scored as the average of five centro-parietal sites (Cz, CP1, CPz, CP2, Pz) and defined as the mean amplitude within a time window between 400 and 1,000 ms following feedback (Hajcak et al., 2009; Weinberg and Hajcak, 2010).

### Data Analysis

All statistical analyses were performed using SPSS Statistics for Microsoft Windows (version 24.0). See Supplementary File 1 for the data underlying the results. To examine changes in mood after the PASAT, PANAS scores from before the PASAT were compared to after processing the PASAT using paired *t* tests. Furthermore, associations of the PANAS with PASAT scores were examined using bivariate correlation analyses using Pearson correlation coefficient. Additionally, it could be assumed that a better PASAT performance would be associated with fewer incorrect trials and therefore with less negative feedback. In turn, the mere difference in the presentation frequency of good vs. bad performers could lead to a differential neural reaction to negative feedback and we would not know if an association of the valence-specific neural activation (Δ = negative-positive feedback) and the PASAT performance could just occur due to this difference and not due to differences in cognitive control functions. Therefore, we additionally calculated the correlation of the number of incorrect trials and the PASAT performance (number of correct trials). To analyze a differential neural activation after correct vs. incorrect trials in the feedback-neutral PASAT we performed paired *t*-tests separately for the FRN, P300 and the LPP. To analyze a differential neural activation to positive vs. negative feedback we performed paired *t*-tests separately for the FRN, P300, and the LPP. To further analyze associations of the valence-specific neural activation (Δ = negative-positive feedback) and changes in the affect ratings with the PASAT performance (number of correct trials and performance stability) we calculated bivariate correlation analyses using Pearson correlation coefficient. For all analyses, two-tailed tests were used, and a 0.05 level of significance was employed.

## Results

### Changes in Affect and Behavioral Data

After the PASAT, overall affect deteriorated significantly as indicated by the PANAS: positive affect ratings decreased [before: *M* = 29.13, *SD* = 5.06; after: *M* = 26.21, *SD* = 5.67; *t*_(23)_ = 2.41, *p* = 0.025] and negative affect ratings increased [before: *M* = 13.29, *SD* = 2.93; after: *M* = 20.96, *SD* = 9.51; *t*_(23)_ = −4.86, *p* < 0.001]. There were no significant correlations of the affect ratings with the PASAT performance (all *p* ≥ 0.472). Furthermore, there was no significant correlation of the number of incorrect trials and the PASAT performance (number of correct trials) [*r*_(22)_ = −0.089, *p* = 0.681]. Concerning the PASAT performance, on average, participants gave 113.04 (*SD* = 31.32) correct, and 52.42 (*SD* = 19.68) incorrect responses with 239.54 (*SD* = 31.45) trials overall (including trials without a response).

### Electrophysiological Data

#### Feedback Related Negativity

**FIGURE 1:**
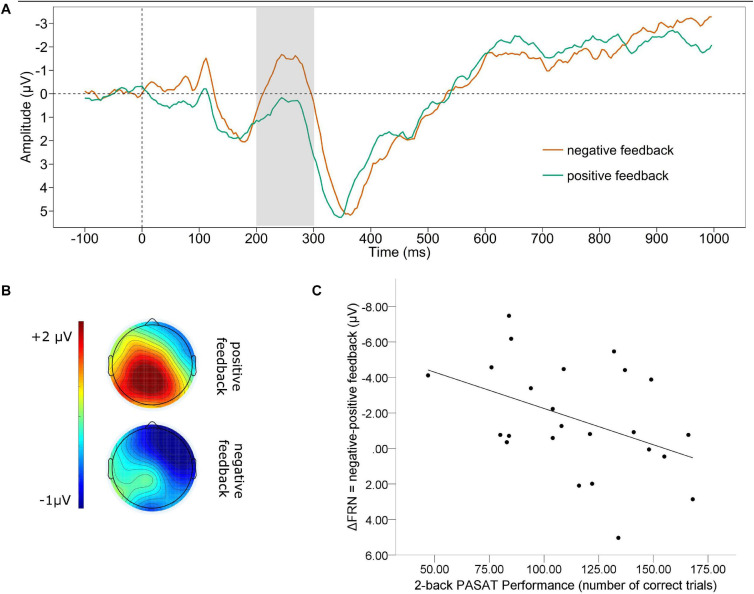
Feedback related negativity. **(A)** Grand average difference wave (PASAT – CP) separately for negative and positive feedback at Fz. **(B)** Scalp map displaying the mean voltage distribution for negative and positive feedback separately (200–300 ms post feedback). **(C)** Scatterplot displaying the PASAT performance as a function of the valence-specific FRN (ΔFRN = negative-positive feedback). Note, for the ΔFRN, more negative values indicate a larger amplitude by negative than positive feedback. Therefore, negative values are at the top of the *Y*-axis.

Figure 1 displays the grand average waveform (A) of the FRN and the mean voltage distribution across the scalp (B) for negative and positive feedback separately (note that higher negative values indicate a larger FRN). The mean amplitude FRN for negative feedback was significantly larger (*M* = −0.817, *SD* = 3.223) than for positive feedback [*M* = 0.846, *SD* = 2.843; *t*_(23)_ = 2.671, *p* = 0.014]. The correlation analysis for the valence-specific neural activation of the FRN (ΔFRN = negative-positive feedback, e.g., a more negative value indicates that the FRN for negative feedback was larger than for positive feedback), revealed a significant association between the ΔFRN and the number of correct trials in the PASAT [see Figure 1C, note that for all scatterplots the *Y*-axis is ordered ascendingly according to the values indicating larger ERPs, e.g., for the FRN values are ordered from positive to negative]. A smaller ΔFRN (e.g., more positive ΔFRN) was linked to a larger amount of correct trials over all [*r*_(22)_= 0.425, *p* = 0.038]. In addition, we found a significant correlation of the ΔFRN and the performance stability. A smaller ΔFRN (e.g., more positive ΔFRN) was linked to a higher performance stability [*r*_(22)_ = 0.433, *p* = 0.034].

#### P300

**FIGURE 2:**
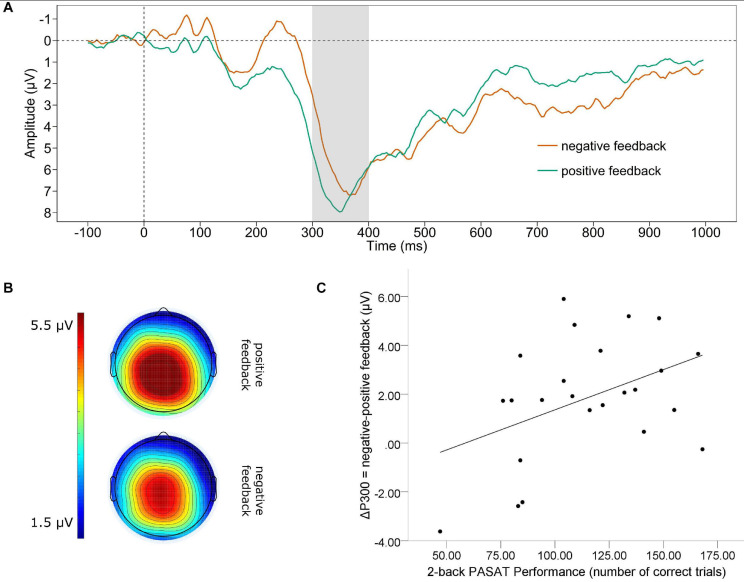
P300. **(A)** Grand average difference wave (PASAT – CP) of the P300 separately for negative and positive feedback averaged across Cz, CPz, Pz. Note that a base-to-peak analysis was performed for the P300. **(B)** Scalp map displaying the mean voltage distribution for negative and positive feedback separately (300–400 ms post feedback). **(C)** Scatterplot displaying the PASAT performance as a function of the valence-specific P300 (ΔP300 = negative-positive feedback).

Figure 2 displays the grand average waveform of the P300 (A) and the mean voltage distribution across the scalp (B) for negative and positive feedback separately. Note that to avoid carry over effects of the shifts in the waveform in the time range of the FRN to the P300, we conducted base-to-peak analyses to define the P300 amplitudes. A paired *t*-test revealed a significant difference between the P300 for positive and negative feedback. The P300 was significantly larger for negative feedback (*M* = 10.648, *SD* = 4.047) than for positive feedback [*M* = 8.812, *SD* = 3.464; *t*_(23)_ = 3.64, *p* = 0.001]. For the valence-specific neural activation of the P300 (ΔP300 = negative-positive feedback), we found a significant correlation between the ΔP300 and the number of correct trials in the PASAT [see Figure 2C]. We observed that a larger P300 elicited by negative as compared to positive feedback (e.g., a more positive ΔP300) was linked to more correct trials [*r*_(22)_ = 0.422, *p* = 0.040]. In addition, we found a significant correlation of the ΔP300 and the performance stability [*r*_(22)_ = 0.465, *p* = 0.022]. A larger P300 by negative as compared to positive feedback was linked to increases in performance stability.

#### Late Positive Potential

**FIGURE 3:**
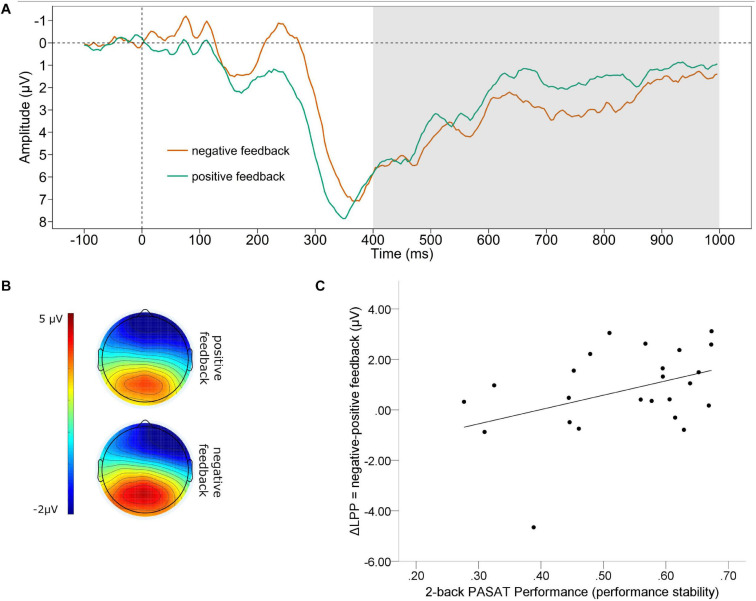
Late positive potential. **(A)** Grand average difference wave (PASAT – CP) of the LPP separately for negative and positive feedback averaged across Cz, CPz, Pz, CP1, CP2. **(B)** Scalp map displaying the mean voltage distribution for negative and positive feedback separately (400–1,000 ms post feedback). **(C)** Scatterplot displaying the PASAT performance as a function of the valence-specific LPP (ΔLPP = negative-positive feedback).

The grand average waveform of the LPP (A) and the mean voltage distribution across the scalp (B) for negative and positive feedback separately, are depicted in Figure 3. We found a significant difference between the mean amplitude LPP for positive and negative feedback. The LPP for negative feedback was significantly larger (*M* = 3.125, *SD* = 3.118) compared to positive feedback [*M* = 2.368, *SD* = 3.026; *t*_(23)_= 2.215, *p* = 0.037]. Further, there was a medium effect sized correlation of the valence-specific neural activation of the LPP (ΔLPP = negative-positive feedback) and the number of correct trials, which failed to reach significance [*r*_(22)_ = 0.300, *p* = 0.155]. However, we found a significant correlation of ΔLPP and performance stability in the PASAT [*r*_(22)_ = 0.407, *p* = 0.049, see Figure 3C]. A larger LPP by negative as compared to positive feedback was linked to increases in performance stability.

### Control Task Feedback-Neutral PASAT

Moreover, there was no difference in neural responses after errors and correct trials in the control task feedback-neutral PASAT for the FRN [correct trials *M* = −0.857, *SD* = 1.125, incorrect trials *M* = −0.633, *SD* = 1.624, *t*_(23)_ = −0.872, *p* = 0.392], P300 [correct trials *M* = 5.550, *SD* = 2.029, incorrect trials *M* = 5.607, *SD* = 2.348, *t*_(23)_ = −0.154, *p* = 0.879] nor LPP [correct trials *M* = 0.399, *SD* = 1.191, incorrect trials *M* = 0.329, *SD* = 1.641, *t*_(23)_ = −0.219, *p* = 0.828]. These results indicate that potential differences in neural reactions to feedback are not due to error monitoring processes but feedback valence (descriptive data, waveforms, and scalp maps for the ERPs of the feedback-neutral PASAT can be found in Supplementary Table 2 and Supplementary Figures 7–9 of the Supplementary Materials).

## Discussion

In this study, we examined electrophysiological characteristics of cognitive control processes and their relation to task performance by means of a challenging and adaptive PASAT. The main findings are (a) that positive and negative feedback induce a differential neural activation throughout the time course of feedback processing (b) that the valence-specific neural activation (negative-positive feedback) is associated with the PASAT performance, and (c) that the direction of this association is critically dependent on the stage of feedback processing.

We found that negative information in the form of performance feedback produced similar neural signals for the FRN time range as in common studies investigating feedback processing (Gehring et al., 2012). Thus, in line with our hypothesis, the FRN was larger for negative feedback than for positive feedback. This observation for the FRN is frequently interpreted as a stronger neural reaction of the anterior cingulate cortex (ACC) for negative than for positive feedback. Since the posterior medial frontal cortex including the ACC is known to reflect the motivational value of stimuli (Ridderinkhof et al., 2004) this further suggests that in early stages of feedback processing in the PASAT, negative feedback is probably perceived as more salient than positive feedback. This makes sense, concerning the fact that negative feedback contains important information to adapt behavior according to changing task demands. Therefore, it could be assumed that a more pronounced reaction to errors is beneficial for task performance. In accordance with this assumption several authors describe a beneficial effect of larger FRN and error-related negativity amplitudes on task performance (Frank et al., 2005; Cohen et al., 2007; Unger et al., 2012; Meyer et al., 2014). For example, when learning a sequence of button presses by trial and error the FRN was significantly larger for trials that were followed by a correct response indicating that a larger FRN was associated with a better learning efficacy (Van Der Helden et al., 2010). However, in our study we found that a larger valence-specific FRN amplitude was associated with poorer task performance, indicating that in the PASAT the feedback plays a different role compared to common studies investigating the FRN. To understand this, it must be considered that in the present study, besides of its informational value, the negative feedback had also the potential to fundamentally distract from task performance, since it was presented simultaneously with the next target. Therefore, we interpret the FRN as a neural signature of attention allocation toward a distractive negative information as opposed to the task relevant target. This is in line with the assumption that the FRN indicates the emotional impact of negative expectation violation (Luu et al., 2003). In accordance with the well-established evidence of a negativity bias linked to a decreased cognitive control in depression, it has been shown that the FRN is enhanced in patients suffering from current as well as remitted depression indicating a hypersensitivity to loss, punishment or negative related stimuli in depression (Tucker et al., 2003; Santesso et al., 2008; Cavanagh et al., 2011) which reflects reduced cognitive control over emotions. This is consistent with our finding of a poorer PASAT performance (number of correct trials as well as the performance stability) in healthy participants with larger FRNs. Moreover, this finding suggests that a larger neural activation following negative than positive feedback is linked to an enhanced sensitivity to negative stimuli, which leads to increased distraction, by the valence-specific neural activation in this early stage of feedback processing.

For the P300 we could also confirm our hypothesis of a stronger neural activation for negative than for positive feedback. Consistent with findings showing that the P300 reflects attention allocation toward motivationally and/or emotionally relevant content, this indicates that negative feedback in the PASAT is associated with greater resource allocation than positive feedback. This assumption is bolstered by the correlation of the ΔP300 (negative-positive feedback) and the PASAT: in contrast to the FRN a larger P300 to negative than positive feedback was associated with a larger number of correct trials and a higher performance stability. This finding is in accordance with previous studies showing comparable associations. For instance it has been observed that a better performance in an n-back working memory task was associated with a larger P300 amplitude (Daffner et al., 2011; Saliasi et al., 2013). Moreover, a larger P300 was found to be associated with more remembered stimuli of emotional content (Palomba et al., 1997). Therefore, our finding adds further evidence that the additional recruitment of neural activity at this stage of processing leads to performance gains and the maintenance of goal-oriented behavior.

In line with our hypothesis, we also found a larger amplitude for negative than for positive feedback for the LPP. Since a large body of evidence shows that the LPP is larger for emotional than for non-emotional stimuli this indicates that negative feedback was perceived as emotionally more relevant than positive feedback (Cacioppo et al., 1996). The fact that negative feedback captures more resources than positive feedback reflected by the LPP suggests that in later stages of feedback processing a negativity bias can be observed. Regarding the valence-specific neural activation of the LPP (ΔLPP = negative-positive feedback) we could observe a similar pattern as for the ΔP300. Although the medium effect sized correlation of the ΔLPP with the number of correct trials in the PASAT failed to reach significance, we found a significant correlation of the ΔLPP and the performance stability, indicating the same association: a larger LPP by negative than positive feedback was associated with a higher performance stability. Just like the association of the ΔP300 with performance, a stronger neural reaction to negative than positive feedback in later processing stages seems to reflect the recruitment of additional cognitive resources, which increase the effective maintenance of coordinated behavior. Our data are in accordance with results showing a positive relationship between larger LPP amplitudes and task performance. This was observed for example in a delayed working memory task: larger ΔLPPs (negative – positive) evoked by emotional pictures serving as distractors were associated with better task performances (Faehling and Plewnia, 2016). Furthermore, also in an approach avoidance task it was found that larger LPP amplitudes were linked to faster RTs (Bamford et al., 2015). In contrast, there is also a finding of larger LPP amplitudes associated with performance deteriorations, indicating increased engagement with a distracting stimulus (Weinberg and Hajcak, 2011). However, it must be considered that no WM task was used in their study, but a speeded response task, focusing on the investigation of attentional processes and less demanding cognitive functions as opposed to our study. In sum, our results for the LPP and the P300 seem to be in line with the idea of an additional recruitment of cognitive resources by emotional stimuli (González-Garrido et al., 2015), at least in these late stages of feedback processing.

Consistent with previous studies, we found affect ratings significantly decreased after PASAT performance (Holdwick and Wingenfeld, 1999; Lejuez et al., 2003; Plewnia et al., 2015). However, there was no significant correlation between the PANAS affect ratings and the PASAT performance (Plewnia et al., 2015). Nevertheless, their functional association is underlined by the correlation between the evoked potentials indicating emotional processing and task performance. For that matter, the use of self-report questionnaires like the PANAS might be not sufficiently precise to detect latent affect changes.

Taken together it appears that in the early stages of feedback processing (<300 ms following feedback) in the PASAT, less automatic resource allocation toward negative than positive feedback is beneficial for task performance. Whereas in later stages (>300 ms following feedback) this association is inverted: a more extensive neural recruitment following negative feedback is linked with better performance. Conceivably, in bad performers increased early (<300 ms) activation after negative feedback interferes with successful memory updating. Apparently, through largely bottom up driven processing, attentional resources are diverted away from target processing and toward distractive negative information, which is reflected by a large neural response to negative feedback. Accordingly, good performance is associated with the ability to engage top-down control already at early processing stages and maintaining attentional resources to targets and not negative feedback information. Opposingly, in later stages of feedback processing (>300 ms), large valence-specific amplitudes seem to reflect resource allocation toward goal directed task processing, indicating successful implementation of top-down control. Therefore, good performers are apparently capable of using the feedback information in a top down driven manner to achieve goal directed behavior, reflected by a large valence specific neural activation in late processing stages.

Overall, the ERP signatures we found contribute to a better understanding of the neural mechanisms underlying the PASAT and furthermore help to better understand why the PASAT is an efficient cognitive control training and could be a promising, innovative treatment option for patients suffering from depression. Our results could indicate that poor performance is associated with increased sensitivity to negative information in early processing stages and reduced allocation of cognitive resources in later stages. As stated above, depressed patients depict a hypersensitivity to negative feedback and negative information in general. PASAT training may help to reduce this hypersensitivity by implementing cognitive control strategies in early processing stages to cope with the frustration caused by PASAT. At the same time, these activated cognitive resources could lead to an effective use of the feedback information in later processing stages. This hypothesis is supported by findings showing a critical involvement of the dlPFC in the PASAT performance (Lazeron et al., 2003), which in turn is a neuronal structure underlying cognitive control functioning and has been found to be hypoactive in depressed patients (Siegle et al., 2007). Our results provide useful tools to test such possible training mechanisms and to determine which patients can benefit from a cognitive control training in the long run.

There are some limitations of the current study. It could be assumed that a better performance in the PASAT would be confounded by fewer incorrect trials and therefore less negative feedback. This would indicate that a differential neural reaction to negative vs. positive feedback could be a result of this difference as opposed to be a marker of cognitive control functions. However, due to the adaptive design of the PASAT, a good performance goes along with a faster stimulus presentation and as a result, participants make more mistakes. This is also reflected by the missing association of the PASAT performance and the amount of negative feedback: good performers receive as much negative feedback as bad performers. In addition, there have been a lot of misses in the task (e.g., trials without a response) that were excluded from data analysis. Probably also these misses reflect meaningful information since they could reflect distraction by negative feedback information. However, during the experiment we observed that the cause for the misses are manifold: participants simply processed the digits not fast enough; sometimes there actually was a response, but it occurred at the same time the feedback was presented (meaning it was not recorded) or sometimes participants zoned out and did not process the stimuli at all for several trials. Unfortunately, we cannot distinguish between these cases afterward but at the same time it can be assumed that their informative value for cognitive functions and neural responses to feedback are quite different. Thus, we decided to exclude misses completely from the analysis. Moreover, it could be possible that differences of neural activation after negative feedback from positive feedback are not due to feedback valence but monitoring processes of behavior based on the mistake. However, if neural activity differences between correct and incorrect trials were based on error as opposed to feedback processing, these differences should also occur in the feedback-neutral PASAT. Yet, this was not the case, indicating that the observed results are due to feedback valence and not error monitoring. Furthermore, to avoid confounds of neural activations of the CP by previously learned associations of color and feedback, the order of performance of the CP and the PASAT was not counterbalanced but the same for all participants (CP first). Therefore, we cannot exclude that also the timing of the measurement affected the results.

To conclude, by elucidating the neural mechanisms underlying the PASAT performance, we demonstrate that enhanced neural activity in early processing stages of negative feedback indicates a diversion of cognitive resources toward negative information resulting in reduced goal-oriented behavior. In turn, additional allocation of resources after salient negative information as indicated by a higher P300 and LPP is linked with enhanced performance and may thus represent a neural signature of successful cognitive control of distractive negative information. Our results provide the basis for further studies using and investigating the PASAT as an effective cognitive control task. Based on these results, future studies will further elucidate associations and malleability of negative information processing, cognitive performance and mood regulation in sensitive population groups and psychiatric disorders.

## Data Availability Statement

The original contributions presented in the study are included in the article/Supplementary Material, further inquiries can be directed to the corresponding author.

## Ethics Statement

The studies involving human participants were reviewed and approved by the Ethics Committee of the Medical Faculty of the Eberhard-Karls-University and at the University Hospital Tübingen. The patients/participants provided their written informed consent to participate in this study.

## Author Contributions

AS and CP conceived and designed the experiments. AS and LE collected the data. AS and LE prepared and processed the electrophysiological data. AS, LE, and CP performed the statistical analysis. AS, LE, and CP wrote the manuscript. All authors contributed to the article and approved the submitted version.

## Conflict of Interest

The authors declare that the research was conducted in the absence of any commercial or financial relationships that could be construed as a potential conflict of interest.
