# Supplementary material for: Neural Signatures of Performance Feedback in the Paced Auditory Serial Addition Task (PASAT): An ERP Study
Source: Front Hum Neurosci. 2021 Feb 18;15:630468. doi: 10.3389/fnhum.2021.630468 (PMC7930379; doi:10.3389/fnhum.2021.630468)
Supplement: Supplementary file 2 [file Table_2.DOCX]

Demographic and neuropsychological Characteristics of the sample

To assess neuropsychological characteristics of the participants, we measured complex attention, motor speed, visual-motor conceptual screening and executive functions with the Trail Making Test (Reitan, 1992). As a measure for approximate verbal intelligence we conducted the Multiple Choice Vocabulary Test (Lehrl, 1995). In addition, we measured participant’s working memory with a short version of a digit span test of the Wechsler Adult Intelligence Scale (Wechsler, 2008). In our digit span task participants had to memorize 2-8 digits (two trials per level of difficulty, both were used for the calculation of the score) and repeat them in the same order (digit span same order) or in the reverse order (digit span reverse order).

**Supplementary Table 1 - Demographic and neuropsychological characteristics of the sample**

| **Characteristic** | **%** | **M** | **SD** | **Range** |
| --- | --- | --- | --- | --- |
| Sex (female) | 66.66 |  |  |  |
| Age |  | 23.71 | 4.06 | 20-37 |
| University entrance diploma (yes) | 95.83 |  |  |  |
| TMT-A (s) |  | 23.12 | 7.55 | 14-51 |
| TMT-B (s) |  | 50.28 | 12.51 | 28-78 |
| Digit span same order |  | 8.33 | 1.95 | 5-12 |
| Digit span reverse order |  | 7.38 | 2.20 | 4-12 |
| MWT-B |  | 66.82 | 21.44 | 26.2-100 |
| **Notes.** TMT-A = time to complete the Trail Making Test part A; TMT-B = time to complete the Trail Making Test part B; Digit span same order = number of correct remembered digits in the Digit span same order task; Digit span reverse order = number of correct remembered digits in the Digit span reverse order task; MWT-B = percentile rank in the Multiple Choice Vocabulary Test. | | | | |

Lehrl, S., Triebig, G., and Fischer, B. (1995). Multiple choice vocabulary test MWT as a valid and short test to estimate premorbid intelligence. *Acta Neurol. Scand.* 91, 335–345. doi:10.1111/j.1600-0404.1995.tb07018.x.

Reitan, R. M. (1992). *Trail Making Test: Manual for Administration and Scoring*. South Tucson, Arizona: Reitan Neuropsychology Laboratory.

Wechsler, D. (2008). *Wechsler Adult Intelligence Scale – Fourth Edition*. San Antonio, Texas: Pearson.

**Supplementary Table 2 – feedback-neutral PASAT**

| **ERP** | **correct trials** | | **incorrect trials** | | **Test statistic** |
| --- | --- | --- | --- | --- | --- |
|  | M | SD | M | SD |  |
| FRN | -0.857 | 1.125 | -0.633 | 1.624 | *t^a^*_(23)_ = -.872, *p* = .392 |
| P300 | 5.550 | 2.029 | 5.607 | 2.348 | *t^a^* _(23)_ = -.154, *p* = .879 |
| LPP | 0.399 | 1.191 | 0.329 | 1.641 | *t^a^* _(23)_ = -.219, *p* = .828 |
| **Notes.** Means (M) and standard deviations (SD) of the amplitudes for the FRN, P300 and LPP separately for correct and incorrect trials in the feedback-neutral PASAT. *^a^*: paired t-Test. | | | | | |

Supplementary Figures 1-9

**
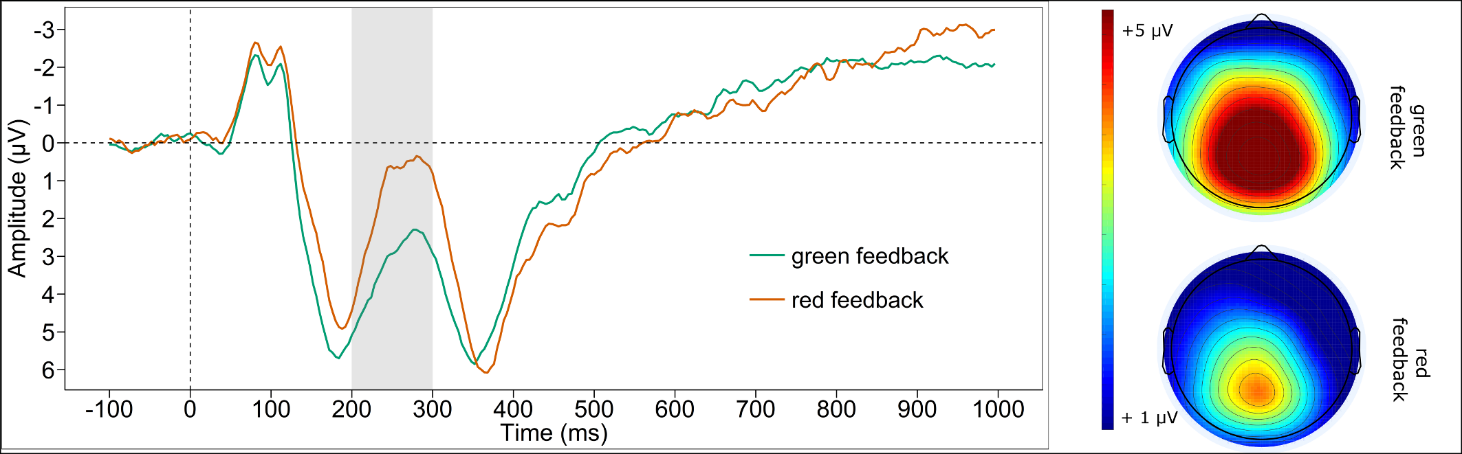
**

**Suppl. Figure 1. Raw FRN for the 2-back PASAT.** The grand average waveform separately for red and green feedback (left panel) at Fz and the scalp map displaying the mean voltage distribution for red and green feedback separately (right panel, 200 - 300 ms post feedback).

**
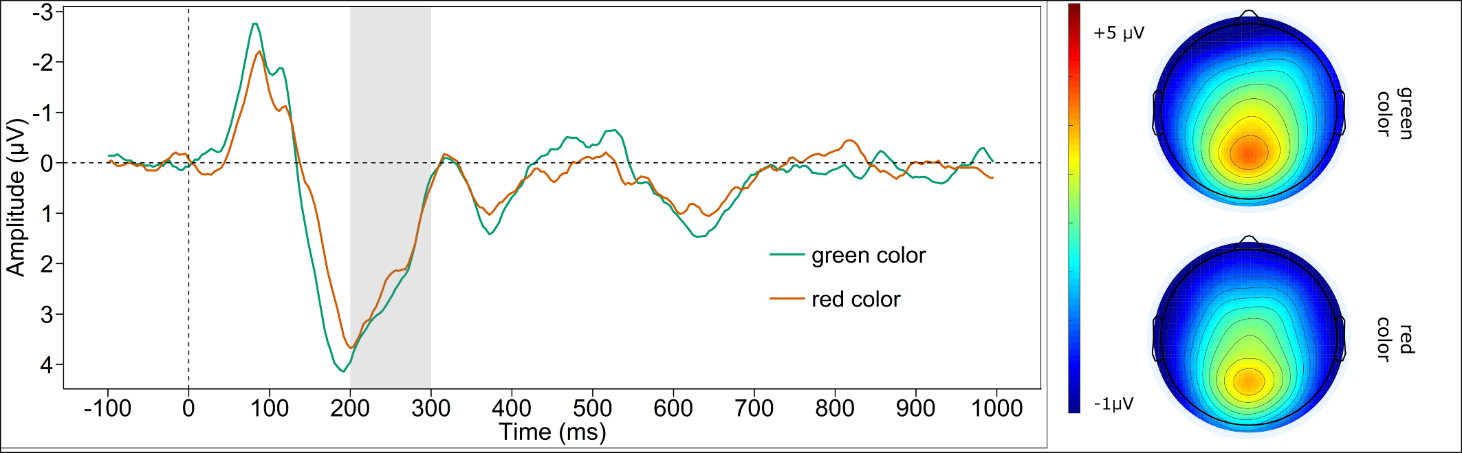
**

**Suppl. Figure 2. Raw FRN for the color presentation task**. The grand average waveform separately for red and green color (left panel) at Fz and the scalp map displaying the mean voltage distribution for red and green color separately (right panel, 200 - 300 ms post color presentation).

**
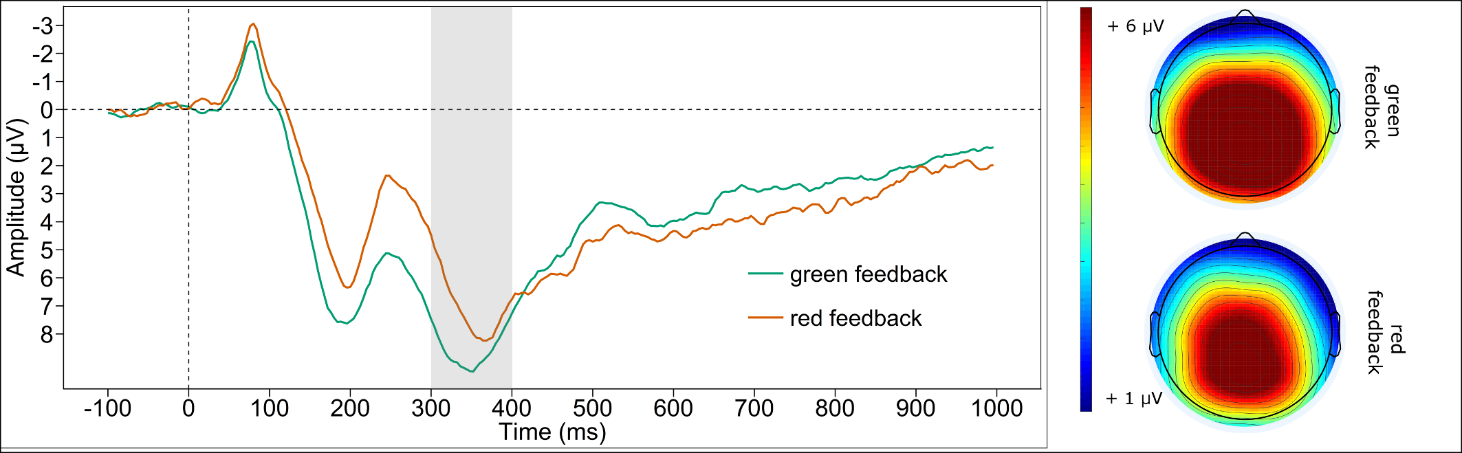
**

**Suppl. Figure 3. Raw P300 for the PASAT**. The grand average waveform separately for red and green feedback (left panel) averaged across Cz, CPz and Pz and the scalp map displaying the mean voltage distribution for red and green feedback separately (right panel, 300 - 400 ms post feedback).

**
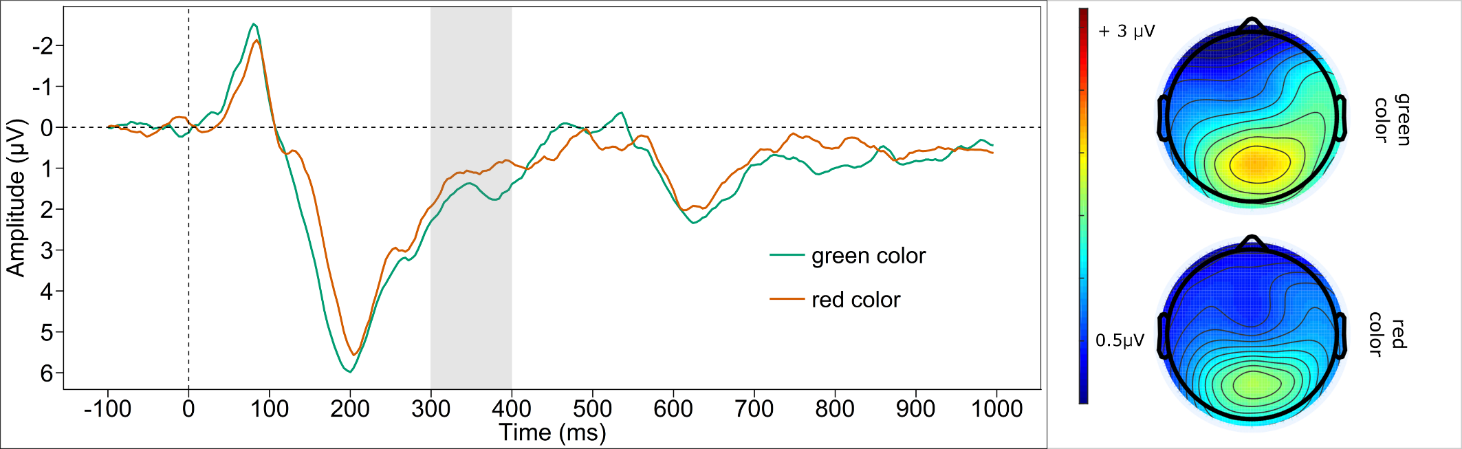
**

**Suppl. Figure 4. Raw P300 for the color presentation task**. The grand average waveform separately for red and green color (left panel) averaged across Cz, CPz and Pz and the scalp map displaying the mean voltage distribution for red and green color separately (right panel, 300 - 400 ms post color presentation).

**
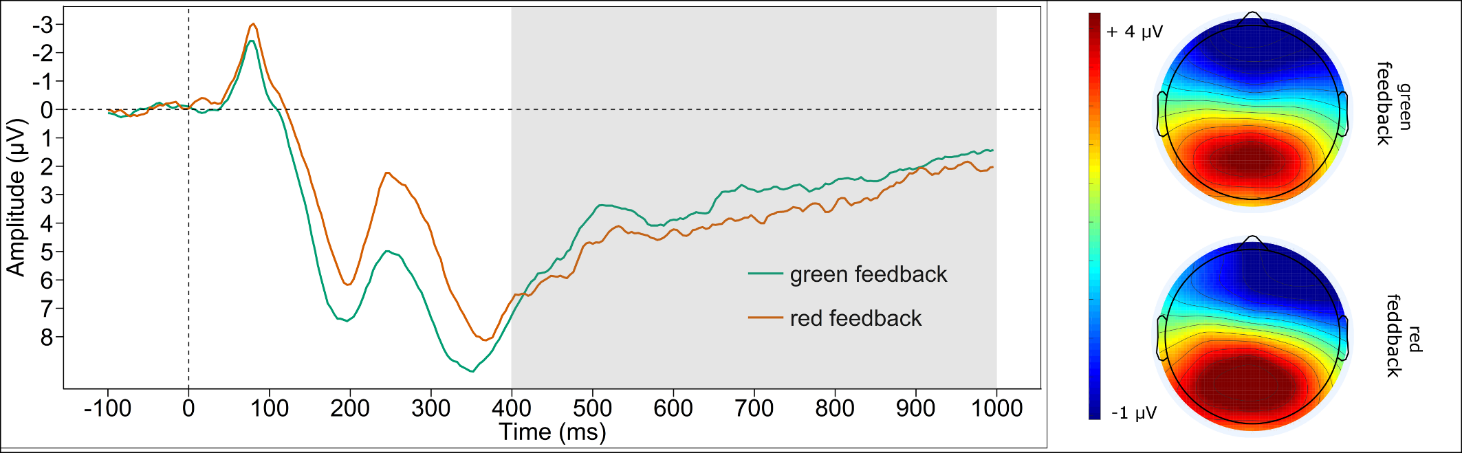
**

**Suppl. Figure 5. Raw LPP for the PASAT**. The grand average waveform separately for red and green feedback (left panel) averaged across Cz, CPz, Pz, CP1 and CP2 and the scalp map displaying the mean voltage distribution for red and green feedback separately (right panel, 400 - 1000 ms post feedback).

**
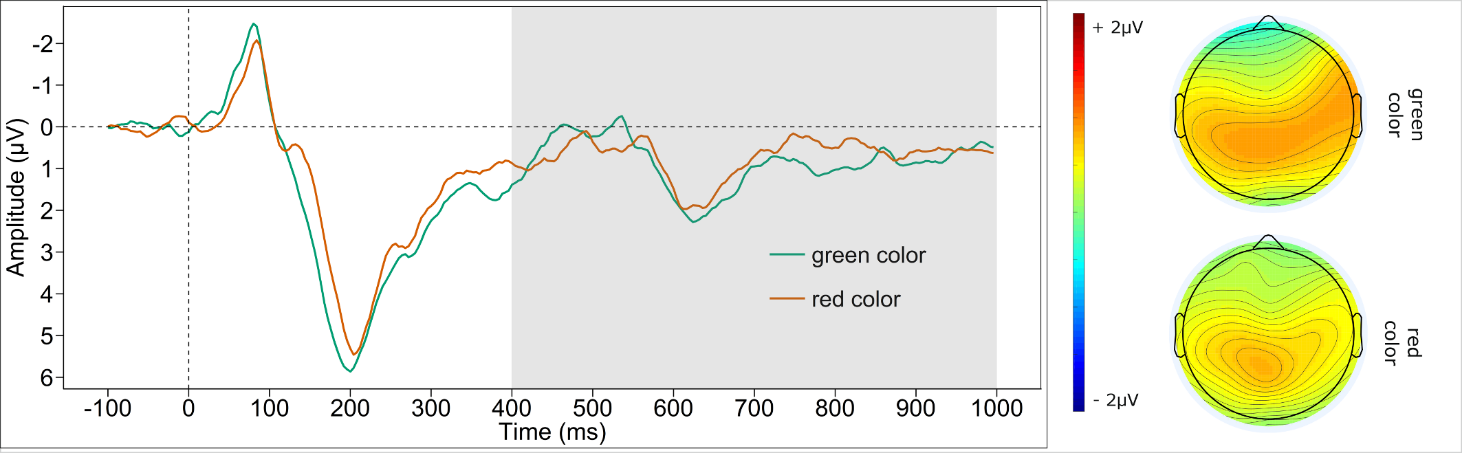
**

**Suppl. Figure 6. Raw LPP for the color presentation task**. The grand average waveform separately for red and green color (left panel) averaged across Cz, CPz, Pz, CP1 and CP2 and the scalp map displaying the mean voltage distribution for red and green color separately (right panel, 400 - 1000 ms post color presentation).

**
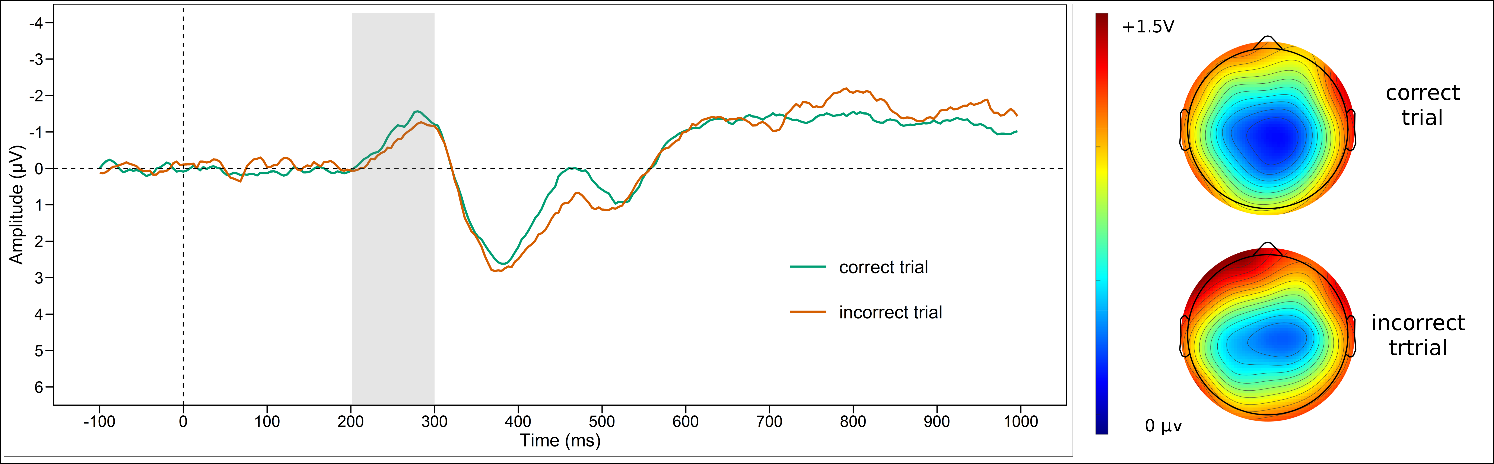
**

**Suppl. Figure 7. Raw FRN for the** **feedback-neutral PASAT.** The grand average waveform separately for correct and incorrect trials (left panel) at Fz and the scalp map displaying the mean voltage distribution for correct and incorrect trials separately (right panel, 200 - 300 ms post digit).


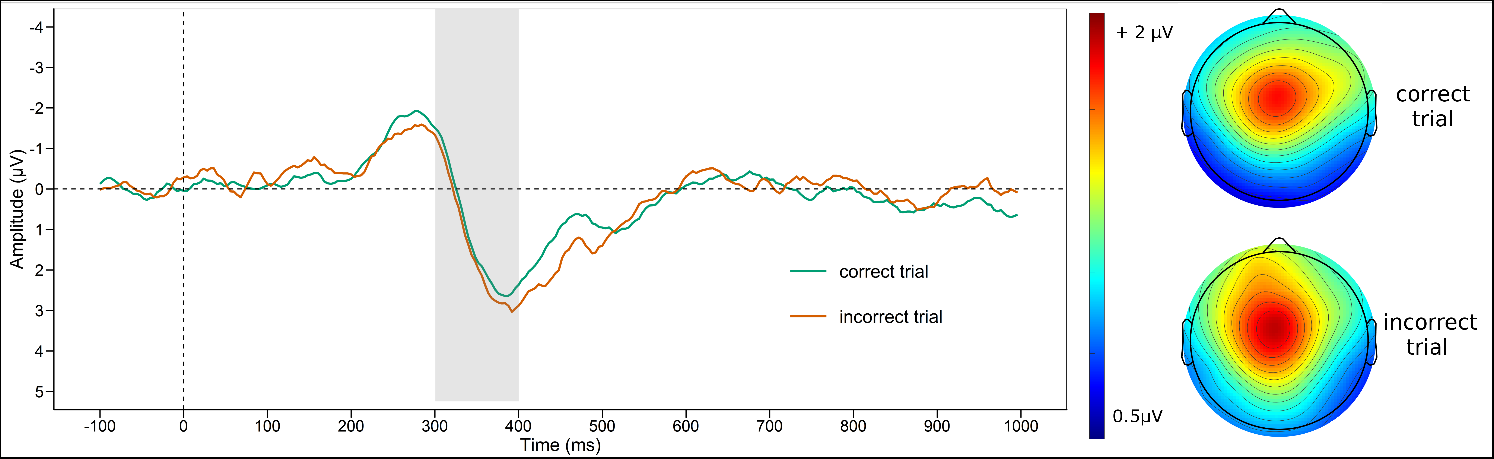


**Suppl. Figure 8. Raw P300 for the feedback-neutral PASAT.** The grand average waveform separately for correct and incorrect trials (left panel) at Cz, CPz, Pz and the scalp map displaying the mean voltage distribution for correct and incorrect trials separately (right panel, 300 - 400 ms post digit).


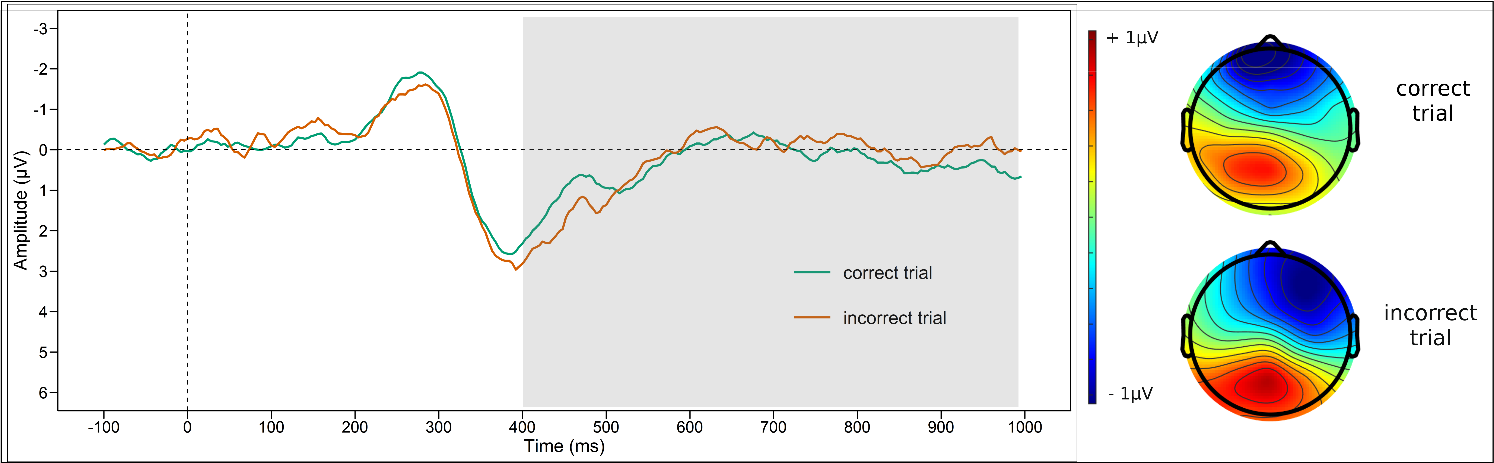


**Suppl. Figure 9. Raw LPP for the feedback-neutral PASAT.** The grand average waveform separately for correct and incorrect trials (left panel) at Cz, CPz, Pz, CP1 and CP2 and the scalp map displaying the mean voltage distribution for correct and incorrect trials separately (right panel, 400 - 1000 ms post digit).
